# The roles of miRNA, lncRNA and circRNA in the development of osteoporosis

**DOI:** 10.1186/s40659-020-00309-z

**Published:** 2020-09-16

**Authors:** Yang Yang, Wang Yujiao, Wang Fang, Yuan Linhui, Guo Ziqi, Wei Zhichen, Wang Zirui, Wang Shengwang

**Affiliations:** 1 People’s Hospital of Guazhou County, Guazhou, 736100 Gansu China; 2 Department of Medical Oncology, Cancer Center, West China Hospital, Sichuan University, Chengdu, 610041 China; 3 Second Clinical Medical College of Lanzhou University, Lanzhou, 730000 China

**Keywords:** Osteoporosis, MiRNA, Lncrna, CircRNA

## Abstract

Osteoporosis is a common metabolic bone disease, influenced by genetic and environmental factors, that increases bone fragility and fracture risk and, therefore, has a serious adverse effect on the quality of life of patients. However, epigenetic mechanisms involved in the development of osteoporosis remain unclear. There is accumulating evidence that epigenetic modifications may represent mechanisms underlying the links of genetic and environmental factors with increased risk of osteoporosis and bone fracture. Some RNAs, such as microRNAs (miRNAs), long non-coding RNAs (lncRNAs), and circular RNAs (circRNAs), have been shown to be epigenetic regulators with significant involvement in the control of gene expression, affecting multiple biological processes, including bone metabolism. This review summarizes the results of recent studies on the mechanisms of miRNA-, lncRNA-, and circRNA-mediated osteoporosis associated with osteoblasts and osteoclasts. Deeper insights into the roles of these three classes of RNA in osteoporosis could provide unique opportunities for developing novel diagnostic and therapeutic approaches to this disease.

## Background

**Fig. 1:**
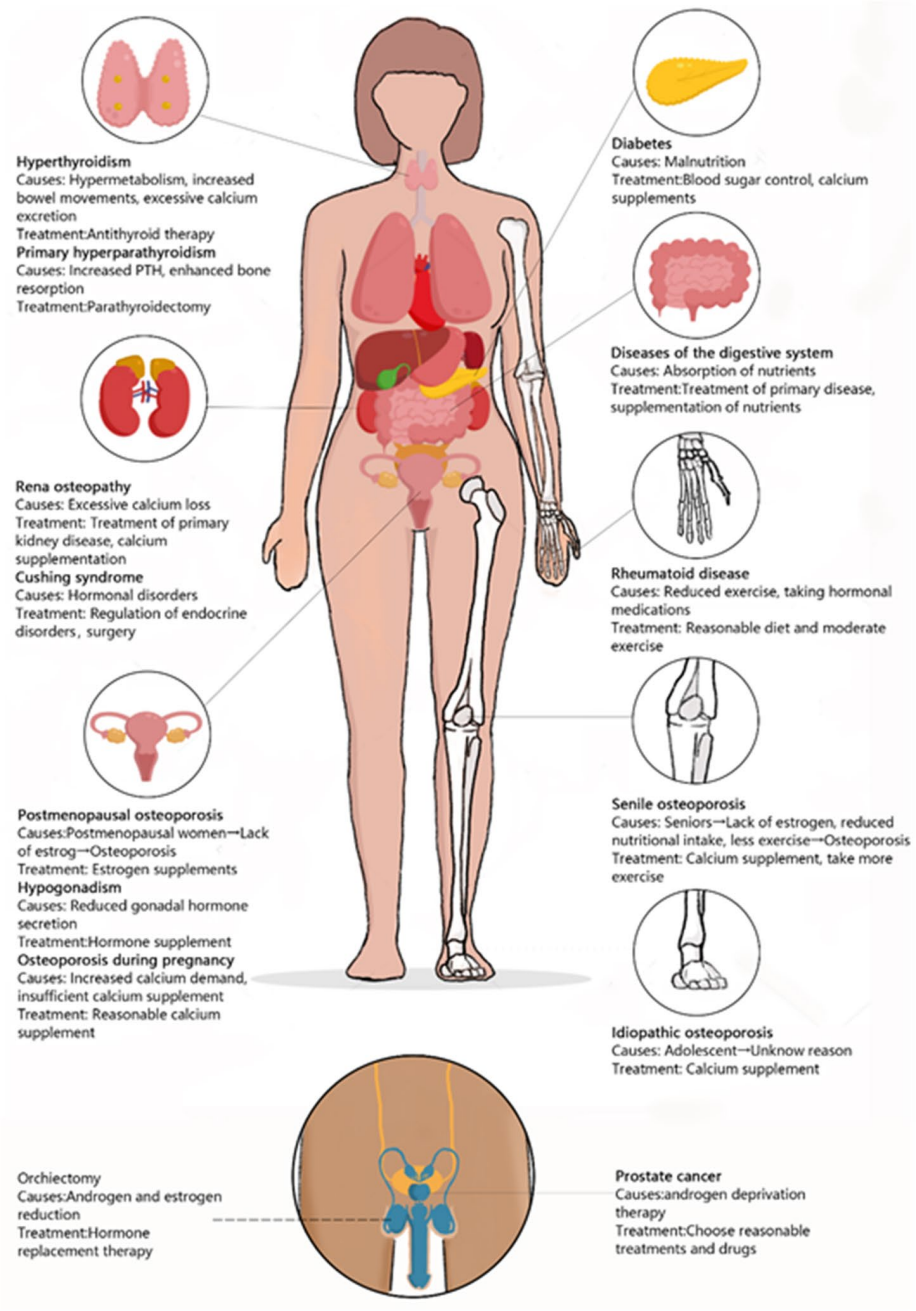
Main causes and diseases of osteoporosis. Main causes of osteoporosis development

Human bone metabolism is a complex process including bone absorption and bone formation mediated by osteoblasts and osteoclasts. Osteoporosis, a metabolic skeletal disease caused by decreased bone formation and increased bone absorption, is characterized by reduced bone mass and deterioration of the microstructure of the bone tissue, and is associated with increased fracture risk [1] (Fig. 1). Previous studies have shown that the occurrence of osteoporosis is related to many factors, including genetic and environmental factors. Most of these factors influence the development of osteoporosis by interfering with osteoblast and osteoclast differentiation and activity. As a widespread and complex disease, the incidence of osteoporosis is increasing markedly with the aging of the population [2]. Osteoporosis is a chronic disease that has adverse effects on quality of life, morbidity, and even mortality. It is estimated that more than 75 million people have osteoporosis worldwide [3]. At present, the approved treatments for osteoporosis mainly include selective estrogen receptor modulators, bisphosphonates, denosumab, and teriparatide, etc. But these methods have limited efficacy as well as numerous adverse effects, and there is as yet no highly effective treatment for osteoporosis [4, 5]. This disease is a major global health problem that places a heavy burden on patients and society. However, recent studies of epigenetics have provided insights that may lead to the possibility of osteoporosis treatment.

**Fig. 2:**
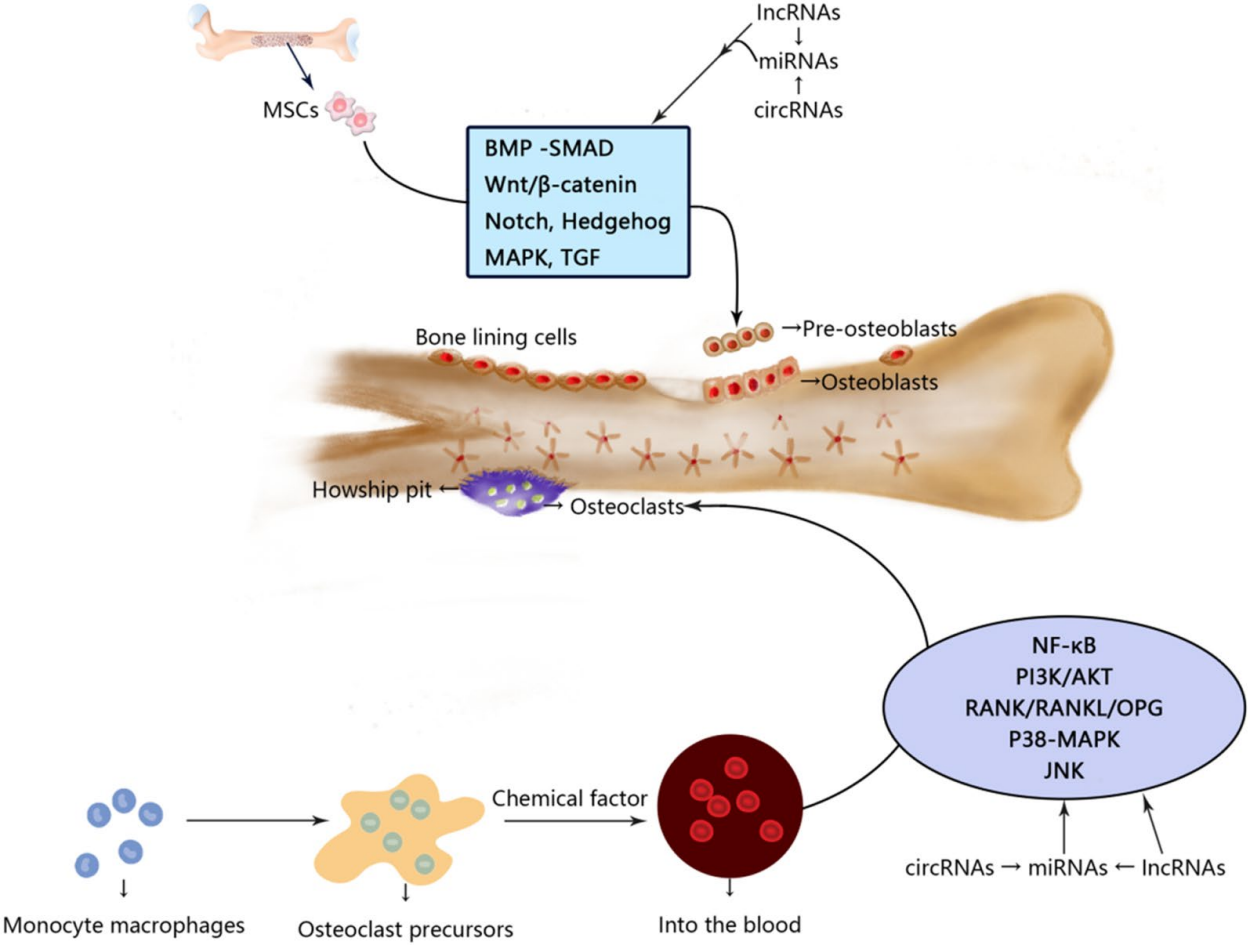
miRNA, lncRNA and circRNA affect osteoblasts and osteoclasts and participate in the development of osteoporosis

Epigenetics is the regulation of gene expression without changes to the underlying DNA sequence [6], and includes DNA methylation, histone modifications, and RNA-based mechanisms [7]. These modifications are expected to result in variable expression of identical genetic information based on the surrounding conditions leading to enhanced expression or silencing of genes [8]. With the in-depth study of epigenetics, there is accumulating evidence that microRNAs (miRNAs), long non-coding RNAs (lncRNAs), and circular RNAs (circRNAs) are closely related to the occurrence and development of various diseases, including inflammatory diseases, metabolic diseases, and cancer [9, 10]. One study had shown that 13 miRNAs, 70 lncRNAs and 260 circRNAs were differentially expressed in patients with postmenopausal osteoporosis (OP group) compared with healthy controls (NC group) [11]. As a multifactorial disease, osteoporosis is closely related to epigenetics. As potential therapeutic targets or biomarkers, there is increasing research and clinical interest in miRNAs, lncRNAs, and circRNAs. The main goal of this review was to set out the roles of miRNAs, lncRNAs, and circRNAs in the occurrence and development of osteoporosis, as related to their effects on osteoblasts and osteoclasts, to provide a theoretical basis for exploring the pathogenesis and potential for clinical treatment of the disease (Fig. 2).

### miRNAs, lncRNAs, circRNAs

#### miRNAs

The miRNAs are small (~ 22 nucleotides), single-stranded, non-coding RNAs encoded by endogenous genes, which have important regulatory effects in cells. miRNAs bind to complementary sequences in their target mRNAs, thereby suppressing expression of the target mRNAs through posttranscriptional regulation, forming a complex regulatory network [12, 13].

It has been speculated that miRNAs regulate one-third of human genes and participate in a number of vital life processes [14].

miRNA-based gene therapy is a rapidly developing disease treatment strategy with a number of advantages. Such therapeutic strategies have great potential for the treatment of osteoporosis.

### lncRNAs

The lncRNAs, which are large non-coding RNAs > 200 nucleotides in length, play important roles in various activities of life [15], including dose compensation effects, epigenetic regulation, and regulation of cell differentiation. Abnormalities of lncRNAs could cause disease, and many studies have shown that lncRNAs are closely related to the mechanism underlying the pathogenesis of osteoporosis.

### circRNAs

The circRNAs, first identified in the cytoplasm of mammalian cells, are more durable than linear RNAs as they have a stable loop structure that prevents exonuclease-mediated degradation [16]. It has been demonstrated circRNAs possess abundant miRNA binding sites, and can act as miRNA sponges. Thus, circRNAs play an essential regulatory role in disease through interactions with disease-related miRNAs [17].

### Mechanisms of action of these three types of RNAs in regulation of osteoblast differentiation in osteoporosis

**Fig. 3:**
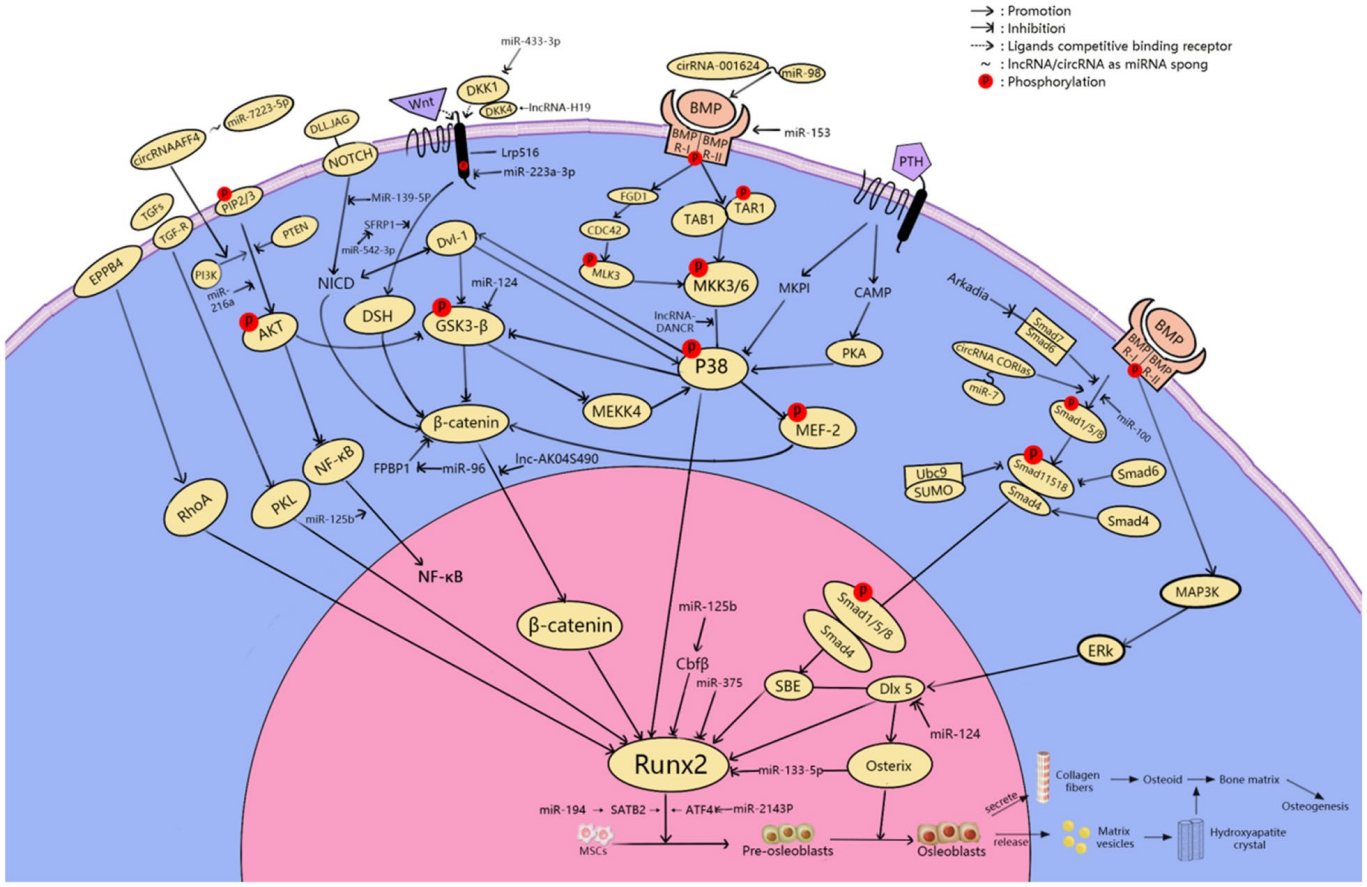
Schematic diagram of the mechanism of miRNA, lncRNA and circRNA affecting osteoblasts in osteoporosis

Osteoblasts are mainly differentiated from mesenchymal progenitor cells in the inner and outer periosteum and bone marrow. These cells specifically secrete a variety of biologically active substances. In bone tissue, a number of signaling pathways are involved in the regulation of bone homeostasis by promoting bone formation [18]. For example, after stimulating the Wnt/β-catenin signaling pathway, glycogen synthase 3 activity decreased and β-catenin phosphorylation and proteasome degradation were inhibited. Therefore, phosphorylated β-catenin collected in the cytoplasm and were then translocated to the nucleus to deactivate gene transcription. This induced the proliferation and differentiation of osteoblasts and reduced apoptosis of mature osteoblasts [19]. As the foremost functional cells for bone formation and reconstruction, osteoblasts are responsible for the synthesis, secretion, and mineralization of bone matrix. Therefore, the differentiation and activity of osteoblasts are particularly closely connected to the occurrence and development of osteoporosis. Here, we review the mechanisms by which miRNAs, lncRNAs, and circRNAs mediate osteoblast differentiation. Adjusting these related molecules may be helpful in the treatment of osteoporosis (Fig. 3).

### miRNA-mediated mechanisms

Some miRNAs have been shown to promote the differentiation of osteoblasts in osteoporosis by regulating the substances related to their differentiation. These miRNAs have an inhibitory effect on the progression of osteoporosis. For example, miR-33-5p acts as a mechanosensitive microRNA that positively regulates osteoblastogenesis by repressing high mobility group AT-hook 2 (Hmga2), which is an inhibitor of osteoblastogenesis [20]. As a key transcription factor, Runt-related transcription factor 2 (Runx2) acts as a modulator regulating the differentiation of mesenchymal stem cells (MSCs) into osteoblasts, which further mature into osteocytes [21]. miRNA-194 promotes osteoblast differentiation by regulating Runx2 nuclear translocation mediated by signal transducer and activator of transcription (STAT1) [22]. As an important biomarker of osteoporosis, Dickkopf-1 (DKK1) is an antagonist of the WNT signaling pathway, and elevation of its level is related to osteolytic bone disease. miRNA-433-3p inhibits the expression of DKK1 protein, activates the Wnt/β-catenin signaling pathway, and promotes osteoblast differentiation [23]. Some groups have suggested that miR-96 inhibits the phosphorylation of epidermal growth factor receptor (EGFR), extracellular signal-regulated kinase 1 (ERK1), and AKT through the downregulation of Human Proheparin-Binding EGF-like Growth Factor (HB-EGF) induced by binding to the 3′ untranslated region (UTR) of its mRNA, thus promoting osteogenic differentiation [24]. In bone tissue, canonical Wnt-signaling is a major regulator of bone homeostasis by promoting bone formation [25]. Another experimental study suggested that miR-96 can activate the Wnt signaling pathway to promote osteogenic differentiation. Animal experiments showed that the level of alkaline phosphatase (ALP) activity, number of calcium nodules, and osteoblast viability increased when the expression level of miR-96 was increased [26]. Besides miR-96, the downregulation of microRNA-139-5p was also reported to positively regulate the Wnt/β-catenin signaling pathway by inducing Notch1 signaling and eventually affecting osteogenic differentiation [27]. The PI3K/AKT pathway is another important pathway in bone metabolism, and miR-216a was shown to regulate the c-Cbl-mediated PI3K/AKT pathway, thereby preserving osteogenesis and enhancing osteoblast differentiation and bone formation [28].

Besides the promoting effects of several miRNAs outlined above, overexpression of some miRNAs could also inhibit osteoblast differentiation and make healing of osteoporosis(it refers to the reduction of symptoms and the increase of BMD) difficult. miR-125b inhibits osteogenic differentiation. On the one hand, it was shown to attenuate osteoblast differentiation of periodontal ligament cells (PDLCs) by targeting NKIRAS2 and enhancing NF-κB signaling [29]. On the other hand, miR-125b was reported to regulate the osteogenic differentiation of human bone marrow-derived mesenchymal stem cells (hBMSCs) by targeting BMPR1b [30]. In the early stages of osteoblast differentiation, miR-125b acts indirectly on Runx2 by targeting a putative binding site for the 3′-UTR of the Cbfβ gene, a key transcription factor for osteogenesis, thereby inhibiting its differentiation [31]. Besides miR-125b, both miR-375 and miRNA-133a-5p could target Runx2. However, miR-375 suppresses osteogenic differentiation by directly targeting Runx2 and, in this process, it reduces the activity levels of key osteoblast markers, including osteocalcin, ALP, and collagen type I α1 (COL1A1) [32]. miRNA-133a-5p inhibits the expression of Runx2, at both mRNA and protein levels by targeting the 3′-UTR of Runx2 [33]. As an endogenous attenuator of Smad1, miR-100 was shown to inhibit BMP-induced osteoblast differentiation [34]. Similarly, miR-203a-3p was reported to suppress osteoblast differentiation by regulating smad9, and the Wnt3a/β-catenin signaling pathway was activated following miR-203a-3 inhibition [35]. Transfer of osteoclast-derived exosomal miR-214-3p to osteoblasts inhibited bone formation [36], which was mediated by inhibition of the protein expression of an important osteogenic transcription factor, ATF4, a target of miR-214-3p in osteoblasts [37]. miR-153, a mechanosensitive miRNA, was shown to regulate osteoblast differentiation by directly targeting BMPR2 [38].

Furthermore, miR-124 and miR-542-3p showed dual-directional regulation of osteoblast differentiation. Their roles in osteoporosis would differ depending on the specific physiopathological state. First, miR-124 was shown to promote the proliferation and differentiation of osteoblasts via the BMP/TGF-β signaling pathway [39]. Moreover, miR-124 was reported to directly target the 3′-UTRs of Dlx3, Dlx5, and Dlx2, which are negative regulators of osteogenic differentiation and bone formation in vivo [40]. Downregulation of miR-124 expression was reported to enhance the expression of GSK-3β, a serine/threonine kinase that can regulate cell differentiation, and then attenuate the activity of the Wnt/β-catenin signaling pathway inhibiting differentiation of osteoblasts [41]. miR-542-3p shows a dual role in osteogenic differentiation. On the one hand, miR-542-3p was shown to play an important positive role in bone formation by inhibiting expression of the secreted frizzled-related protein-1 (SFRP1), a negative regulator of the WNT signaling pathway, and to induce osteoblast differentiation [42]. On the other hand, overexpression of miR-542-3p caused repression of BMP-7 and inhibition of BMP-7/PI3K survival signaling, which would limit osteogenic differentiation and promote osteoblast apoptosis [43].

Based on the above findings, some groups have proposed that comparing miRNA levels in serum before and after osteoporosis treatment would further verify their mechanism of action in mediating osteoporosis. After 3 months of treatment with TBTD, miR-33 expression was downregulated, and serum levels of miR-133a were reduced after 12 months of treatment. Although miR-124 level did not change significantly with TPTD treatment, the level of miR-124 expression was lower after the third month of treatment and the responsiveness of bone mineral density (BMD) to TPTD treatment was higher at the twelfth month [44]. Other studies showed that teriparatide and zoledronate could regulate miRNA levels in the treatment of osteoporosis. For example, they increased the expression of miR-203a-3p in the tissues and serum of untreated ovariectomized animals [45]. In summary, miRNAs are closely related to osteoblast differentiation. Therefore, these specific miRNAs may be useful therapeutic targets for targeted medication, and it may be possible to treat osteoporosis by adjusting their content and expression levels in patients. Furthermore, miRNAs transported through vesicles can serve as diagnostic markers in osteoporosis treatment (Table 1).

**Table 1:** The role of some miRNAs in osteoporosis and their mechanisms through osteoblasts and osteoclasts and targets

miRNA ID	Target molecule and pathway	Mechanisms that affect osteoporosis through osteoblasts and osteoclasts
miR-33-5p [20]	Hmga2	Promote osteoblast differentiation
miR-194 [22]	Runx2	Promote osteoblast differentiation
miRNA-433-3p [23]	DKK1	Promote osteoblast differentiation
miR-96 [24, 26]	EGFR, HB-EGF	Promote osteoblast differentiation Promote osteoblast differentiation
	Wnt/β-catenin signaling pathway	
miR-139-5p [27]	NOTCH1,Wnt/β-catenin pathway	Promote osteoblast differentiation
miR-216a [28]	PI3K/AKT pathway	Promote osteoblast differentiation Promote osteoblast differentiation
	BMP/TGF-β signaling pathway,	
miR-124 [39–41]	GSK-3β, Wnt/β-catenin pathway	Inhibit osteoblast differentiation Inhibit osteoblast differentiation
	Dlx3, Dlx5, and Dlx2	
miR-542-3p [42, 43]	SFRP1	Promote osteoblast differentiation
	BMP-7/PI3K- survivin signaling	Inhibit osteoblast differentiation Inhibit osteoblast differentiation
	NKIRAS2, NF-κB signaling	
miR-125b [29–31]	BMPR1b	Inhibit osteoblast differentiation
miR-375 [32]	Runx2	Inhibit osteoblast differentiation
miR-100 [34]	Smad1	Inhibit osteoblast differentiation
miR-203a-3p [35]	Smad9, Wnt/β-catenin signaling pathway	Inhibit osteoblast differentiation
miR-214-3p [35, 37]	ATF4	Inhibit osteoblast differentiation
miR-153 [38]	BMPP2	Inhibit osteoblast differentiation
miR-21 [69, 70]	RANKL, PI3K/Akt signaling pathway, PDCD4	Promote osteoclast differentiation
miR-183 [71]	RANKL, HO-1	Promote osteoclast differentiation
miR-155 [72, 73]	TNF-α, IL-1β, RANKL, M-CSF, RANK, TRAP, Bcl-2, LEPR, AMPK, p-AMPK, OPG, Bax, TAB 1	Promote osteoclast differentiation
miR-223 [74]	TWIST and Runx2	Promote osteoclast differentiation
miR-19a [74]	TWIST and Runx2	Promote osteoclast differentiation
miR-214 [75]	Pten, PI3K/Akt pathway	Promote osteoclast differentiation
miR-182 [76]	Foxo3, Maml1	Promote osteoclast differentiation
miR-26a [77]	CTGF/CCN2	Inhibit osteoclast differentiation
miR-31 [78]	RhoA	Inhibit osteoclast differentiation
miRNA-17 [79]	RANKL	Inhibit osteoclast differentiation
miR-503 [80]	RANK	Inhibit osteoclast differentiation
miR-126-5p [81, 82]	PTHrP and MMP-13	Inhibit osteoclast differentiation
miR-7b [83]	DC-STAMP	Inhibit osteoclast differentiation
miR-141 [84]	Calcr, EphA2	Inhibit osteoclast differentiation

### lncRNA-mediated mechanisms

A number of lncRNAs have been shown to promote osteoblast differentiation, and it was speculated that they may be useful in the treatment of osteoporosis. First, the histone decarboxylase SIRT1 was shown to be an important positive regulator of osteoblastogenesis and bone mass. However, the expression of SIRT1 was inversely proportional to the expression of lncRNA HIF1A-AS1, suggesting the function of lncRNA HIF1A-AS1 in osteogenic differentiation, although the specific mechanism is not yet clear [46]. Second, lncRNA HoxA-AS3 was reported to interact with Enhancer Of Zeste 2 (EZH2) and to be required for H3 lysine-27 trimethylation (H3K27me3) of the key osteogenic transcription factor Runx2. Therefore, lncRNA HoxA-AS3 is a significant molecule in osteoblast differentiation [47].

In contrast to lncRNA HoxA-AS3, lncRNA-DANCR was shown to recruit enhancer of zeste homolog 2 (EZH2) to promote H3K27me3 by interacting with 305-nt transcript and enhancer of zestehomolog2, ultimately inhibiting transcription of the target gene Runx2 and osteogenic differentiation [48]. Moreover, DANCR also mediated the proliferation and osteogenic differentiation of hBMSCs through inactivation of p38 MAPK [49]. lncRNA ANCR was shown to be a fundamental regulator of osteogenic differentiation; when upregulated, it inhibited osteogenic differentiation by inhibiting signaling pathways [50]. Furthermore, lncRNAs that inhibited osteoblasts in differentiation through the WNT/β-catenin signaling pathway included lncRNA HOTAIR and lncRNA AK045490. lncRNA HOTAIR downregulated the expression of Wnt/β-catenin signaling pathway-related proteins to inhibit signal transduction and inhibited osteoblast differentiation. DKK1 was when to reduce the protein levels of HOTAIR, β-catenin, Cyclin D, C-myc, and Runx2, which could partially reverse the regulatory effect of HOTAIR on Wnt/β-catenin [51]. Moreover, when the expression level of lncRNA p21 is decreased, the Wnt/β-catenin signaling pathway is activated by increased secretion of E2, finally stimulating bone formation and osteogenic differentiation [52]. The DKK4 gene encodes a protein belonging to the Dickkopf family. Downregulation of lncRNA H19 reduced the expression level of Dkk4, thereby inhibiting the Wnt/β-catenin signaling pathway and negatively regulating osteogenic differentiation [53]. lnc-AK045490, which is enriched in skeletal tissues, inhibited osteoblast differentiation and bone formation by inhibiting nuclear translocation of β-catenin and downregulating the expression of TCF1, LEF1, and Runx2 [54]. Similarly, lnc-AK016739 inhibited osteogenic differentiation and bone formation because it could inhibit the expression and activity of osteoblastic transcription factors [55]. Inhibition of lncRNA UCA1 was shown to promote osteoblast proliferation and differentiation by activating the BMP-2/(Smad1/5/8) signaling pathway in osteoblasts [56].

Furthermore, lncRNA MEG3 plays dual roles in the differentiation of osteoblasts, showing both positive and negative regulation. First, lncRNA MEG3 was shown to promote the proliferation and differentiation of osteoblasts by activating the Wnt/β-catenin signaling pathway, so it is expected to become a new target for accelerating fracture healing [57]. However, downregulation of lncRNA MEG3 inhibited osteogenic differentiation by enhancing expression of IGF1 [58]. The observations outlined above suggest that most lncRNAs have inhibitory effects against osteogenic differentiation. Therefore, it may be possible to silence the expression of these specific lncRNAs using specifically targeted drugs, thereby limiting the development of osteoporosis (Table 2).

**Table 2:** The role of some lncRNAs in osteoporosis and their mechanisms through osteoblasts and osteoclasts and targets

LncRNA ID	Target molecule and pathway	Effects on osteoblasts differentiation
lncRNA HIF1A-AS1 [46]	SIRT1	Promote osteoblast differentiation
LncRNA HoxA-AS3 [47]	EZH2, H3K27me3, Runx2	Promote osteoblast differentiation
lncRNA MALAT1 [102]	miR-143, miR-204	Promote osteoblast differentiation
lncRNA MODR [103]	miR-454	Promote osteoblast differentiation
LncRNA KCNQ1OT1 [104]	miR-214	Promote osteoblast differentiation
LncRNA NTF3-5 [105]	miR-93-3p	Promote osteoblast differentiation
LncRNA POIR [106]	miR-182	Promote osteoblast differentiation
LncRNA Linc-ROR [107]	miR-145	Promote osteoblast differentiation
LncRNA H19 [100, 101]	miR-675, miR-141, miR-22	Promote osteoblast differentiation
LncRNA H19 [53]	Wnt/β-catenin pathway,	Inhibit osteoblast differentiation
LncRNA-DANCR [48, 49]	EZH2, H3K27me3, Runx2, p38 MAPK	Inhibit osteoblast differentiation
LncRNA ANCR [50]	Wnt/β-catenin pathway	Inhibit osteoblast differentiation
LncRNA HOTAIR [51]	Wnt/β-catenin pathway	Inhibit osteoblast differentiation
lncRNA p21 [52]	E2, Wnt/β-catenin pathway	Inhibit osteoblast differentiation
Lnc-AK045490 [54]	β-catenin, TCF1, LEF1 and Runx2	Inhibit osteoblast differentiation
Lnc-AK016739 [55]	osteoblastic TF	Inhibit osteoblast differentiation
lncRNA UCA1 [56]	BMP-2/(Smad1//5/8)	Inhibit osteoblast differentiation
LncRNA MEG3 [57, 58]	Wnt/β-catenin signaling pathway	Promote osteoblast differentiation
	IGF1	Inhibit osteoblast differentiation
LncRNA HOTAIR [108]	miR-17-5p, SMAD7	Inhibit osteoblast differentiation
LncRNA MIAT [109]	miR-150-5p	Inhibit osteoblast differentiation
lncRNA-ORLNC1 [111]	miR-296	Inhibit osteoblast differentiation
LncRNA MEG3 [112]	miR-133a-3p	Inhibit osteoblast differentiation
LncRNA TSIX [52]	miR-30a-5p, and Runx2	Promote osteoblast apoptosis
lncRNA TUG1 [86]	PTEN	Promote osteoclast differentiation
lncRNA AK077216 [87]	NIP45, NFATc1	Promote osteoclast differentiation
lncRNA SNHG15 [88]	RANK/RANKL pathyway	Promote osteoclast differentiation
LncRNA-Jak3 [89]	Nfatc1, Ctsk	Promote osteoclast differentiation
LncRNA LINC00311 [90]	DDL3	Promote osteoclast differentiation
LncRNA RP11-498C9.17 [91]	HDAC4	Inhibit osteoclast differentiation
LncRNA Bmncr [92]	RANK	Inhibit osteoclast differentiation
LncRNA NONMMUT037835.2 [93]	RANK, NF-κB/MAPK signaling pathway	Inhibit osteoclast differentiation
LncRNA-NEF [94]	IL-6	Inhibit osteoclast differentiation

### Circular RNA-mediated mechanisms

circRNAs have also been reported to play roles in osteoblast differentiation and thus also participate in the development of osteoporosis. During osteogenesis, circRNA_0016624 was reported to activate miR-98 and enhance BMP2 expression, which is known to play an important role in induction of osteogenic differentiation, and so circRNA_0016624 promoted osteoblast differentiation [59]. Acting as a sponge for miR-7223-5p, circRNA AFF4 promoted MC3T3-E1 cell proliferation and inhibited apoptosis under the action of PI3KR1 [60]. circRNA CDR1as was shown to be an inhibitor of miR-7, which upregulated GDF5 expression, and stimulated Smad1/5/8 and p38 MAPK phosphorylation, thereby promoting osteogenic differentiation of PDLSCs [61]. Studies have shown that circHIPK3 expression was reduced in necrotic bone tissue, and miR-124 was upregulated after targeted knockout of circHIPK3, thereby increasing osteoblast cell death. Dexamethasone- and H_2_O_2_-induced cytotoxicity in osteoblasts was/were reported to be associated with downregulation of circHIPK3 [62, 63]. In contrast, an inverse correlation was observed between circIGSF11 and miR-199b-5p. It found that this correlation was upregulated during the osteogenesis of hBMSCs. Silencing of circIGSF11 promoted osteoblast differentiation and increased the expression of miR-199b-5p [64]. At present, only the mechanisms of action of the above-mentioned circRNAs in osteoblast differentiation have been determined. There has been less research on circRNAs compared with miRNAs and lncRNAs, and this should be the subject of future studies related to osteoporosis (Table 3).

**Table 3:** The role of some circRNAs in osteoporosis and their mechanisms through osteoblasts and osteoclasts and targets

circRNA ID	Target molecule and pathway	Mechanisms that affect osteoporosis through osteoblasts and osteoclasts
circRNA_0016624 [59]	miR-98, BMP2	Promote osteoblast differentiation
circRNA AFF4 [60]	miR-7223-5p	Promote osteoblast differentiation
CircRNA CDR1as [61]	miR-7, GDF5, Smad1/5/8 and p38 MAPK	Promote osteoblast differentiation
circHIPK3 [62, 63]	miR-124	Promote osteoblast differentiation
circRNA436 [111]	miR-108, miR-335	Promote osteoblast differentiation
CircRNA BANP [112]	miRNA-146a, PDGFRA	Promote osteoblast proliferation
circRNA ITCH [112]	miR-34a, DUSP1	Promote osteoblast proliferation
circIGSF11 [64]	miR-199b-5p	Inhibit osteoblast differentiation
hsa_circ_0127781 [111]	miR-210, miR-335	Inhibit osteoblast differentiation
CircRNA_28313 [95]	miR-195a, CSF1	Promote osteoclast differentiation
CircRNA_007438 [96]	miRNA-6338, miRNA-7028-3p	Promote osteoclast differentiation
circRNA_005108 [96]	miRNA-6975-3p,miRNA-6516-5p, miRNA-486b-5p, miRNA-31-3p	Inhibit osteoclast differentiation

### Mechanisms of action three kinds of RNA in regulation of osteoclast differentiation in osteoporosis

Osteoclasts, derived from blood mononuclear macrophages, are terminally differentiated cells. The principal function of osteoclasts is to mediate bone resorption, and they play an important role in bone development, growth, repair, and reconstruction. Abnormal osteoclast function can cause abnormal bone resorption. Hyperfunction can cause bone degenerative diseases, such as osteoporosis, so osteoclast differentiation plays a significant role in osteoporosis. RANK was osteocalcin receptor of RANKL. RANK recognizes and binds with RANKL on the surface of osteoclasts and osteoclast progenitor cells in a cell type-dependent manner, and has been show to directly promote the differentiation, activation, and maturation of osteoclasts. As a signaling molecule, RANKL effectively induces the generation and differentiation of osteoclasts. Osteoprotegerin (OPG) is a new member of the tumor necrosis factor (TNF) receptor family, known as osteoclastogenesis inhibitory factor (OCIF), so it has the function of inhibiting osteoclasts [65–67]. Therefore, the RANK/RANKL/OPG pathway is very important in bone tissue metabolism [68].

**Fig. 4:**
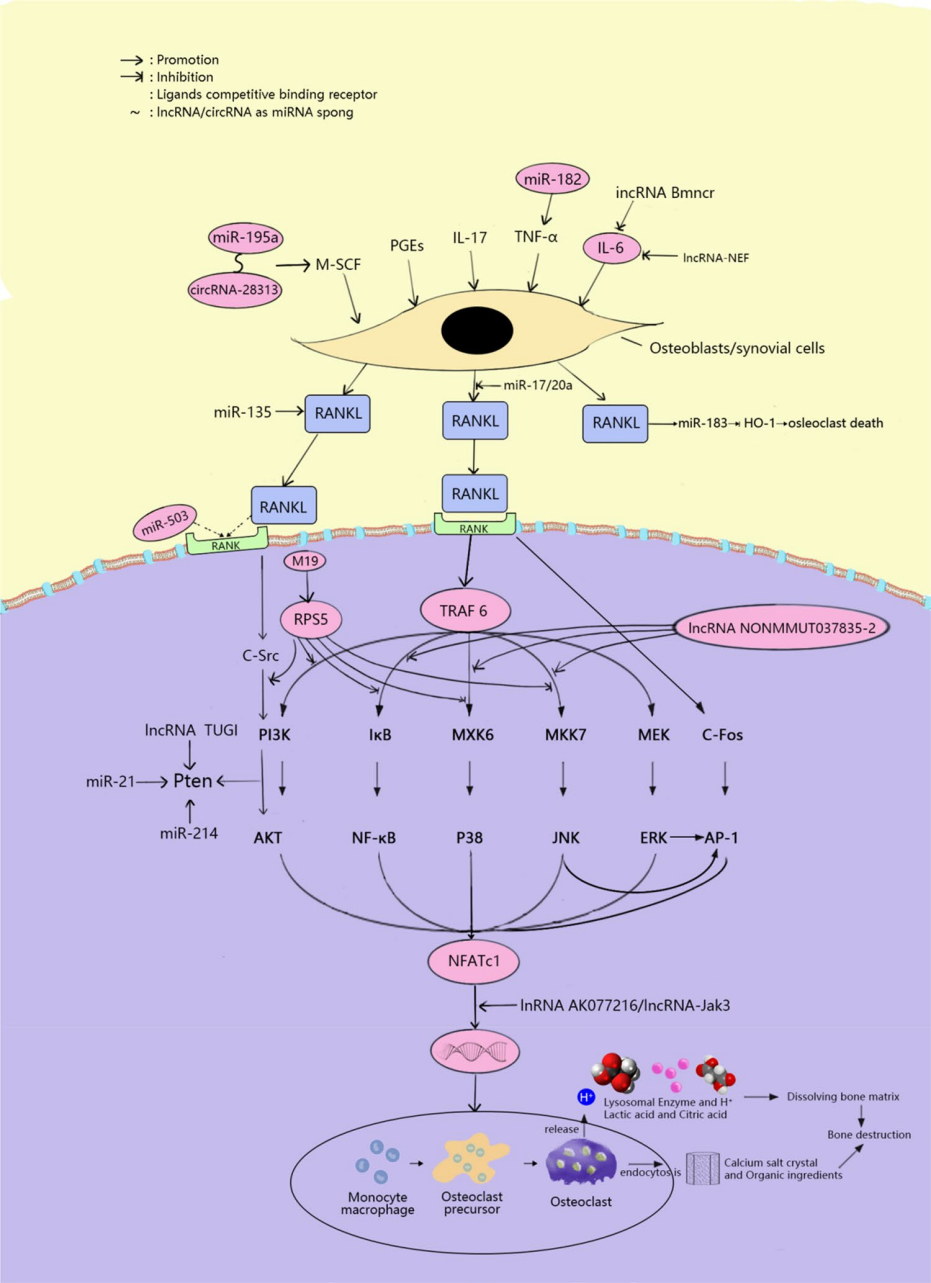
Schematic diagram of the mechanism of miRNA, lncRNA and circRNA affecting osteoclasts in osteoporosis

As mentioned above, anomalous increases in osteoclast number would accelerate the formation and progression of osteoporosis. Previous studies have shown that miRNAs, lncRNAs, and circRNAs affect osteoclast differentiation, and so they also have roles in osteoporosis. Here, we summarized these relevant research results to provide new ideas and a foundation for research and treatment of osteoporosis from the perspective of osteoclasts (Fig.  4).

### microRNA-mediated mechanisms

Here, we discuss miRNAs that could promote the process of osteoclast differentiation. In theory, these miRNAs may have adverse effects on osteoporosis. Previous studies had shown that multiple miRNAs could affect differentiation of osteoclasts induced by RANKL. First, during osteoclastogenesis, miR-21 could be upregulated by RANKL and may promote osteoclastogenesis and bone resorption by targeting Pten to activate the PI3K/Akt signaling pathway or targeted downregulation of programmed cell death protein 4 (PDCD4) [69, 70]. Second, increased RANKL-induced miR-183 can inhibit heme oxygenase-1 (HO-1) by binding to its 3′-UTR, thereby positively regulating osteoclastogenesis [71]. Third, downregulation of miR-155 can decrease TNF-α, IL-1β, RANKL, M-CSF, RANK, TRAP, and Bcl-2 expression, and increase LEPR, AMPK, p-AMPK, OPG, and Bax expression, thus inhibiting osteoclast proliferation and bone resorption [72]. Lipopolysaccharide (LPS)-induced miR-155 promoted autophagy to increase osteoclast formation by decreasing Table 2 expression [73]. Fourth, miR-223 and miR-19a regulated the expression of Twist and Runx2, affected the expression of RANK/RANKL pathway and MCP-1, and ultimately regulated the pathophysiology of osteolytic bone destruction [74]. Fifth, as a key regulator of skeletal metabolism, on the one hand, miR-214 promoted osteoclast activity by targeting Pten via the PI3K/Akt pathway. On the other hand, it also mediated osteoclast–osteoblast crosstalk through exocrine miRNA paracrine mechanisms [75]. Finally, miR-182 promoted TNF-α-induced osteoclastogenesis by inhibiting Foxo3 and Maml1, and this process was controlled by RBP-J [76]. The above findings suggested that drugs may affect the development and treatment of osteoporosis by affecting the expression of the above-mentioned miRNAs. In contrast, the miRNAs discussed below can inhibit osteoclast differentiation by targeting related molecules, and so may allow the development of innovative methods for treating osteoporosis. RANKL was shown to upregulate the expression of miR-26a at the late stage of osteoclastogenesis. A number of miR-26a inhibitors can enhance RANKL-induced osteoclast formation and CTGF expression. Connective tissue growth factor/CCN family 2 (CTGF/CCN2) was shown to promote osteoclast formation by suppressing the expression of dendritic cell-specific transmembrane protein (DC-STAMP). Its expression could be downregulated by miR-26a mimic in osteoclast precursor cells resulting in the inhibition of osteoclast formation, actin ring formation, and bone resorption [77]. Second, miR-31 was shown to inhibit RANKL-induced osteoclast formation by regulating RhoA expression (RhoA plays an important role in the regulation of cytoskeleton remodeling) [78]. Third, miR-17/20a targeting reduces the expression of RANKL in osteoclasts; thus, it has an inhibitory effect on osteoclast differentiation [79]. Finally, RANK is the target of miR-503, which can bind to RANKL and induce osteoclast differentiation and activation, and silencing of miR-503 in CD14^+^ PBMCs promotes osteoclastogenesis [80]. The RANK/RANKL/OPG pathway is closely connected with osteoclasts, but miRNA can also regulate osteoclast differentiation via other mechanisms. miR-126-5p is an intronic miRNA that can control osteoclast differentiation by negatively regulating PTHrP and MMP-13 [81, 82]. miR-7b was reported to inhibit osteoclastogenesis and cell fusion by directly targeting DC-STAMP [83]. miR-141 has been identified as a quintessential negative inhibitor of osteoclastogenesis and bone resorption. The functional mechanism involves its targeting of two osteoclast differentiation factors, calcitonin receptor (Calcr) and ephrin A receptor 2 precursor (EphA2) [84].

Recent experiments have shown that oleanolic acid, a new drug that can be used to treat osteoporosis, inhibits RANKL-induced osteoclastogenesis through the ERα/miR-503/RANK signaling pathway in RAW264.7 cells [85]. This represents an example of successful osteoporosis treatment based on miRNA. With the exception of miR503, the above studies demonstrated that these miRNAs are important signaling molecules in the RANK/RANKL/OPG pathway and may serve as therapeutic targets for the prevention and treatment of osteoporosis (Table 1).

### lncRNA-mediated mechanisms

lncRNAs have been shown to regulate osteoclastogenesis by regulating the expression of specific target mRNAs. In osteoporosis, the increased expression of lncRNA TUG1 can regulate the proliferation and apoptosis of osteoclasts through Pten [86]. During the formation of osteoclasts, the expression of lncRNA AK077216 was significantly up-regulated. This lncRNA inhibits the expression of NIP45 and promotes the expression of NFATc1, a major transcription factor involved in osteoclast differentiation [87]. Downregulation of lncRNA SNHG15 expression suppressed osteoclasts by regulating the RANK/RANKL pathway [88]. lncRNA-Jak3-mediated Nfatc1 activation was reported to upregulate cathepsin K (Ctsk) expression to promote MSU-induced osteoclast differentiation [89]. lncRNA LINC00311 promoted osteoclast differentiation by inhibition of DDL3 expression and regulating the Notch signaling pathway [90].

Of course as expected, lncRNAs also have inhibitory effects on osteoclasts. The expression levels of these lncRNAs were inversely associated with the severity of osteoporosis. lncRNA RP11-498C9.17 was closely related to various epigenetic regulatory factors, such as HDAC4, MORF4L1, HMGA1, and DND1. Among them, downregulation of histone deacetylase was shown to promote osteoclast differentiation, suggesting that lncRNA RP11-498C9.17 may regulate osteoclast production through HDAC4 [91]. lncRNA Bmncr is also a negative regulator of RANKL-induced osteoclast differentiation [92]. IL-6 intervened in regulation of bone mineral density and osteoclast differentiation and activation. lncRNA-NEF may play a role in osteoporosis by inhibiting IL-6. Studies in postmenopausal osteoporosis showed that a high lncRNA-NEF level was associated with a substantially reduced course of treatment and lower post-treatment recurrence rate [93]. lncRNA NONMMUT037835.2 regulated osteoclastogenesis by negatively regulating RANK expression and inhibiting the NF-κB/MAPK signaling pathway. Upregulation of lncRNA NONMMUT037835.2 inhibited osteoclast differentiation [94].

In summary, these lncRNAs that had effects on osteoclast differentiation may represent a breakthrough in the treatment of osteoporosis. Decreases and increases in the lncRNA expression levels promoted and suppressed osteoclast differentiation, respectively (Table 2).

### circRNA-mediated mechanisms

So far, most studies had focused on miRNAs, and there were few studies on the relationship between circRNAs and osteoclasts. Among them, the research on circRNA_28313 was relatively clear. It relieved miR-195a-mediated CSF1 inhibition by acting as competing endogenous RNA (ceRNA). miR-195a directly targeted circRNA_28313 and CSF1 3′-UTR, and could form a ceRNA network to regulate RANKL + CSF1-induced osteoclast differentiation in BMM cells [95]. circRNA_007438 was upregulated during osteoclastogenesis and targeted miRNA-6338 and miRNA-7028-3p. In contrast, circRNA_005108 was downregulated during osteoclastogenesis and targeted miRNA-6975-3p, miRNA-6516-5p, miRNA-486b-5p, and miRNA-31-3p [17]. Further studies of the relationship between circRNA and osteoclast differentiation will be very useful for the treatment of osteoporosis (Table 3).

### Interactions between three miRNAs

Previous studies showed that miRNAs could lead to gene silencing by binding to mRNAs, and competitive endogenous RNAs could regulate gene expression by competitively binding with miRNA response elements (MREs). Therefore, the ceRNA hypothesis revealed a novel mechanism for inter-RNA interactions [96]. Both the circRNA and lncRNA involved in our review had miRNA binding sites, which acted as miRNA sponges in the cells, and then counteracted the inhibitory effects of miRNAs on their target genes, thereby enhancing the levels of target gene expression. This interaction could form a complex ceRNA network, which plays a major role in various biological processes and disease progression. In osteoporosis, some lncRNAs and all circRNAs affected osteoblast and osteoclast differentiation by acting as miRNA sponges. Examination of this interaction will facilitate analysis of the pathogenesis of osteoporosis, and will be advantageous for the development of novel drugs for treating osteoporosis.

### Interactions between lncRNAs and miRNAs

A number of lncRNAs have been shown to interact with miRNAs during osteoblast differentiation [97]. H19/miR-675 was reported to suppress mRNA and protein expression of transforming growth factor-β1 (TGF-β1) and histone deacetylase (HDAC) 4/5. This resulted in inhibition of Smad3 phosphorylation, promoting osteoblast differentiation [98, 99]. As an lncRNA closely related to osteoporosis, H19 can also regulate the Wnt/β-catenin signaling pathway through interactions with miRNAs. miR-141 and miR-22 are negative regulatory factors for osteogenesis and the Wnt/β-catenin signaling pathway. lncRNA-H19 acts as a sponge for these two miRNAs, thus negatively regulating their functions and thereby promoting osteoblast proliferation [98, 100]. lncRNA MALAT1 is also a regulator of osteogenic differentiation via two mechanisms. First, lncRNA MALAT1 was reported to regulate osterix overexpression in human MSCs, and then bind to and inhibit miR-143 to promote osteogenic differentiation. Second, lncRNA MALAT1 acts as a sponge for miR-204 and increases the expression of Smad4, which activates and supports the expression of ALP and osteocalcin, thereby promoting bone formation and mineralization [101]. As a molecular sponge for miRNAs, lncRNA MODR binds to miR-454 and mitigates its inhibitory effect on Runx2, thereby promoting bone formation [49]. lncRNA KCNQ1OT1 acts as a ceRNA and interacts directly with miR-214, upregulating the expression of BMP2 and osteogenic genes in bone marrow MSCs [49]. lncRNA NTF3-5 was shown to downregulate miR-93-3p, thereby promoting osteogenic differentiation and bone regeneration [102]. lncRNA POIR competes for miR-182 binding sites resulting in downregulation of its target gene, FOXO1. FOXO1 inhibits the classic Wnt signaling pathway by competing with TCF4 for β-catenin, thereby promoting bone formation. In particular, the NF-κB pathway was shown to be abnormally activated during inflammation, thereby increasing the expression level of miR182 and reducing the level of lncRNA POIR, disrupting the balance of the lncRNA-POIR-miR-182 regulatory network and eventually affecting bone formation [103]. lncRNA Linc-ROR as a sponge for miR-138 and miR-145 antagonizes the functions of these two miRNAs (both of which are negative regulators of osteogenesis) and inhibits their shared target, ZEB2, eventually activating Wnt/β-catenin signaling pathway, which enhances osteogenesis [104].

Besides promoting effects, this interaction was also shown to inhibit osteoblast differentiation. lncRNA HOTAIR downregulation causes decreases in the expression levels of miR-17-5p and, consequently, of the miR-17-5p target, SMAD7. After si-lncRNA HOTAIR, the activity of RUNX2, COL1A1 mRNA expression and ALP would be preeminent [105]. lncRNA MIAT could regulate its binding to target genes by sponging miR-150-5p, and its overexpression inhibits osteogenic differentiation [106]. lncRNA-ORLNC1 acts as a ceRNA for miR-296, reducing the ability of this miRNA to promote osteoblast differentiation by targeting Pten [49]. lncRNA MEG3 regulates the expression of miR-133a-3p, and inhibits osteogenic differentiation of BMSCs obtained from postmenopausal women with osteoporosis [107]. lncRNA TSIX was shown to downregulate the expression of miR-30a-5p, and Runx2 expression was suppressed by knockdown of TSIX. Thus, lncRNA TSIX promoted apoptosis of osteoblasts by downregulating miR-30a-5p [108].

Most studies regarding the relationship between lncRNA and miRNA concentrated on the effects on osteogenic differentiation, and there have been few related studies on osteoclasts. One study showed that, during osteoclastogenesis, lncRNA-AK131850 can act as a sponge for miR-93-5p in newborn and mature osteoclasts, increasing vascular endothelial growth factor A (VEGF-A) transcription, expression, and secretion by reducing miR-93-5, thereby promoting vasculogenesis of endothelial progenitor cells [109]. Although this finding was not directly related to osteoporosis, it was closely related to osteoclasts, so it may be useful in developing treatments for osteoporosis.

### Interactions between circRNAs and miRNAs

Previous research had showm that Circular RNAs (circRNAs) serve as competing endogenous RNAs (ceRNAs) and indirectly regulate gene expression through shared microRNAs (miRNAs) [110]. The introduction to circRNAs above mentioned the following pairs of interacting circRNAs and miRNAs: circRNA_0016624 and miR-98 [59]; circRNA AFF4 and miR-7223-5p [60]; circRNA CDR1as and miR-7 [61]; and circHIPK3 and miR-124 [62, 63]. All four pairs of interactions promote osteoblast differentiation. However, the interaction of circIGSF11 and miR-199b-5p inhibits osteoblast differentiation [64]. Moreover, miR-107 inhibits the Wnt/β-catenin signaling pathway by downregulating Dkk-1. miR-335 was shown to regulate the osteogenic differentiation of MSCs through the Wnt/β-catenin signaling pathway. Analysis of the interaction network showed that circRNA436 is closely related to both miRNAs. Therefore, circRNA436 may affect the Wnt/β-catenin signaling pathway as one of the key regulators of osteogenic differentiation [111]. circRNA BANP combined with miRNA-146a, which targets platelet—derived growth factor receptor alpha (PDGFRA) and thus regulates osteogenic differentiation, and circRNA ITCH, exerted this effect by combining with miRNA-34a that targets dual-specificity phosphatase 1 (DUSP1) [112].

circRNAs can affect osteoclast differentiation by binding to multiple miRNAs, e.g., circRNA_007438 binds miRNA-6338 and miRNA-7028-3p. Furthermore, circRNA_005108 was shown to bind to the four miRNAs, miRNA-6975-3p, miRNA-6516-5p, miRNA-486b-5p, and miRNA-31-3p, which contributed to inhibition of osteoclast differentiation [52]. It has been demonstrated that circRNAs are closely related with osteoporosis through their effects as miRNA sponges, but these results were limited. Further studies may facilitate the development of novel treatments for osteoporosis.

### Summary and future perspectives

This review described some RNAs with important roles in the differentiation of osteoblasts and osteoclasts and their molecular mechanisms of action. miRNAs, lncRNAs, and circRNAs act by targeting major genes and signaling pathways related to osteoblast and osteoclast differentiation. Moreover, both lncRNAs and circRNAs can be utilized as miRNA sponges. They play important regulatory roles in various signaling pathways through their interactions with each other, through which they affect cell differentiation. The Wnt/β-catenin signaling pathway and its key regulatory molecules, which play important roles in osteoblast differentiation, are targets for multiple regulatory RNAs. The RANK/RANKL/OPG receptor–ligand system is an important element in the differentiation and development of osteoclasts, and these RNAs can act on this system to regulate osteoclast differentiation. The regulation of these two cell types by these three classes of RNAs is essential in the dynamic process of osteoporotic bone metabolism imbalance [52], and so they are intimately related to the development of osteoporosis. Large numbers of miRNAs have been shown to be related to osteoporosis. However, due to the paucity of studies on circRNAs, only a few circRNAs related to osteoblast and osteoclast differentiation have been reported to date.

Osteoporosis has a serious adverse effect on the quality of life of patients and places a major burden on society. Therefore, it is necessary to establish the pathogenesis of osteoporosis and develop effective treatments. As molecules that can regulate the differentiation of osteoblasts and osteoclasts through multiple pathways, miRNAs, lncRNAs, and circRNAs can be used as strategic targets or biomarkers with potential application to the diagnosis and treatment of osteoporosis. Furthermore, a better understanding of the regulation of the expression of these RNAs will be instrumental in the development of explicit targeting approaches to treat osteoporosis.

## Conclusion

This review discussed the mechanisms by which miRNAs, lncRNAs, and circRNAs affect osteoporosis, to help in clarifying the shortcomings of current research. Although there are increasing numbers of studies on epigenetics, and current research has also identified a variety of RNAs related to osteoporosis, there is still room for improvement. The discovery of more meaningful RNAs related to osteoporosis, and determination of their interactions, will facilitate further clarification of the pathogenesis of osteoporosis and the development of more efficient drugs and treatment strategies, which will be beneficial to patients, medical practitioners, and to society as a whole.

## Data Availability

Not applicable.

## Abbreviations

miRNAs: microRNAs
lncRNAs: Long non-coding RNAs
circRNAs: Circular RNAs
Hmga2: High mobility group AT-hook 2
Runx2: Runt-related transcription factor 2
MSCs: Mesenchymal stem cells
STAT1: Signal transducer and activator of transcription
DKK1: Dickkopf-1
EGFR: Epidermal growth factor receptor
ERK1: Extracellular signal-regulated kinase 1
HB-EGF: Human Proheparin-Binding EGF-like Growth Factor
UTR: 3′ Untranslated region
ALP: Alkaline phosphatase
PDLCs: Periodontal ligament cells
hBMSCs: Human bone marrow-derived mesenchymal stem cells
COL1A1: Collagen type I α1
SFRP1: Secreted frizzled-related protein-1
BMD: Bone mineral density
H3K27me3: H3 lysine-27 trimethylation
PDCD4: Programmed cell death protein 4
HO-1: Heme oxygenase-1
EphA2: Ephrin A receptor 2 precursor
ceRNA: Competing endogenous RNA
